# Circular RNA circCRKL inhibits the proliferation of acute myeloid leukemia cells via the miR-196a-5p/miR-196b-5p/p27 axis

**DOI:** 10.1080/21655979.2021.1982310

**Published:** 2021-10-07

**Authors:** Wen Liu, Fanjun Cheng

**Affiliations:** Institute of Hematology, Union Hospital, Tongji Medical College, Huazhong University of Science and Technology, Wuhan, China

**Keywords:** CircCRKL, acute myeloid leukemia, miR-196a-5p, miR-196b-5p, p27

## Abstract

As a new type of non-coding RNA, the role of circular RNA (circRNA) in various diseases and tumors has received considerable attention. Studies have shown that circRNAs play an important role in the progression of acute myeloid leukemia (AML) via different mechanisms. However, the specific underlying molecular mechanism of circRNAs in the proliferation of AML cells remians unclear. This study aimed to clarify the biological role and mechanism of circCRKL in AML. The results indicated low circCRKL expression in AML cell lines and samples. Moreover, the overexpression of circCRKL inhibited the proliferation and colony-forming ability of AML cells, while its silencing promoted them. In addition, bioinformatics tools and luciferase assays revealed that circCRKL could sponge miR-196a-5p and miR-196b-5p to promote the expression of p27. Furthermore, circCRKL inhibited AML cell proliferation via the miR-196a-5p/miR-196b-5p/p27 axis, suggesting a potential new target for AML therapy.

## Introduction

Acute myeloid leukemia (AML) is a heterogeneous and invasive hematological malignancy [1]. Over the last few years, considerable efforts have been made to treat AML. However, more than half of AML patients experience resistance or recurrence [2]. Allogeneic hematopoietic stem cell transplantation has been recognized as the only effective way to cure AML [3]. Therefore, it is essential to understand the AML progression mechanism to develop effective treatment strategies.

Circular RNAs (circRNAs), a new type of non-coding RNAs, exhibit continuous closed loops and stable expression in mammalian cells while reportedly participating in the progression of many diseases by regulating various biological processes, including gene expression and transcription [4–6]. Generally, circRNAs serve as competitive endogenous RNAs (ceRNAs) to regulate downstream pathways via miRNA binding [7]. For example, circ_0008532 has been found to act as an oncogene to promote bladder cancer progression by binding miR-155-5p and miR-330-5p [8]. A recent study has revealed that circ_0119872 promotes the progression of uveal melanoma via miR-622/G3BP1 axis [9]. In AML, some circRNAs have been found to be involved in the progression of the disease [10,11].

V-Crk Avian Sarcoma Virus CT10 Oncogene Homolog-Like (CRKL) (GeneID:1399) gene is reported to be involved in the progression of myeloid leukemia [12,13], whether circCRKL derived from the CRKL gene is involved in the progression of myeloid leukemia remains unclear. In addition, circCRKL is reportedly a tumor inhibitor in prostate cancer by targeting miR-141 [14].

This study aimed to identify a new circRNA/miRNA/mRNA network in AML. Here, we hypothesized that circCRKL suppressed the proliferation of AML cells via miR-196a-5p/miR-196b-5p/p27 axis. CircCRKL may be a promising target in the treatment of AML.

## Materials and methods

### Tissue specimens

Bone marrow (BM) samples were obtained from 34 primary AML (non-acute promyelocytic leukemia) patients and 34 healthy volunteers from the Institute of Hematology of Union Hospital affiliated of Tongji Medical College. The detailed clinical parameters of the AML samples are shown in Table S1. All samples were obtained with the informed consent of the patients and were approved by Union Hospital, Tongji Medical College, Huazhong University of Science and Technology Ethics Committee ([2020]0075–01).

### Cell culture

Human AML cell lines, KG-1a (French American British classification systems-M0 (FAB-M0)), KG-1 (FAB-M1), Kasumi-1 (FAB-M2), and the human BM stromal cell line, HS‐5, were purchased from the American Type Culture Collection (ATCC, VA, USA). THP1 (FAB-M5) and MOLM13 (FAB-M5) [15] were obtained from the China Center for Type Culture Collection (Wuhan, China). The cells were cultured in RPMI 1640 medium (HyClone, USA, Cat no. SH30605.01) containing 10% fetal bovine serum (Gibco, Australia, Cat no. 10,099–141 C) in a humidified incubator at 37°C with 5% CO_2_.

### Quantitative real-time polymerase chain reaction (qRT-PCR)

The total RNA was isolated using Trizol reagent (Takara, Dalian, China, Cat no. 9108). RNase R treatment was processed at 37°C with RNase R (Epicenter, WI, USA, Cat no. RNR07250). Complementary DNA (cDNA) was then directly synthesized and reversed with PrimeScript RT Master Mix (Takara, Dalian, China, Cat no. RR036A). qRT-PCR was performed using the SYBR kit (Takara, Dalian, China, Cat no. RR820A) and analyzed via a StepOne Plus system (Life Technologies, Carlsbad, CA) [16]. GAPDH and U6 were used as control genes for circRNA and miRNA, respectively. The fold changes were calculated through relative quantification (2^−ΔΔCt^). The primer sequences are provided in Table S2.

### Cell transfection

The pCD5‐ciR vector (Geneseed, Guangzhou, China) was used for circCRKL overexpression. The human p27 cDNA was synthesized and inserted into pcDNA3.1 by Vigene (Shandong, China). The shRNAs against circCRKL and p27 were designed and cloned into pGPU6-GFP-Neo (GenePharma, Shanghai, China). The miR-196a-5p/miR-196b-5p mimics, negative control (miR-NC), and anti-miR-196a-5p/anti-miR-196b-5p inhibitor were purchased from RiboBio (Guangzhou, China). The transfection was performed with lipofectamine 3000 reagent (Invitrogen, CA, USA, Cat no. L3000015) as previously described [17]. The detailed sequences are provided in Table S2.

### Cell counting kit-8 (CCK-8) assay

Approximately 5 × 10^3^ cells per well were plated into a 96-well plate (Corning, NY, USA, Cat no. 3599) and incubated at 37°C. After incubation with 10 μL of CCK-8 reagent (DOJINDO Laboratories, Kumamoto, Japan, Cat no. CK04) for 2 h, the cell viability was measured at 450 nm using a microtiter plate reader [18].

### Cell cycle analysis

AML cells (1 × 10^6^) were fixed using 70% cold absolute ethyl alcohol, stained with propidium iodide reagent (BD Pharmingen, USA, Cat no. 550,825), and analyzed using flow cytometry (BD LSRFortessaTM X-20) [16]. ModFit LT software was used to analyze the results.

### Colony-forming assay

The 6-well culture plates were pre-coated with 2 ml of 1.2% agarose gel (BBI Life Sciences, Shanghai, China, Cat no. A600015-0025) mixed with cell culture medium (bottom layer). Then the AML cells (0.3 × 10^4^) were suspended in 1 ml of 0.7% agarose gel mixed with cell culture medium and added to the bottom layer. The surrounding wells were supplemented with 1 ml of phosphate buffered saline (Thermo Fisher Scientific, USA, Cat no. 10,010,031) and placed in an incubator after the solidification of the gel. The clones were stained with MTT (3-(4,5-dimethylthiazol-2-yl)-2,5-diphenyltetrazolium bromide) (Sigma, USA, Cat no. M5655) and scored after 10–14 days [19].

### RNA binding protein immunoprecipitation assay (RIP), circRNA in vivo precipitation (circRIP)

The transfected AML cells were lysed with Radio-Immunoprecipitation Assay (RIPA) buffer (Thermo Scientific, MA, USA, Cat no. 89,900) containing magnetic beads conjugated with anti-Ago2 antibody (Cell Signaling Technology, USA, Cat no. 2897). A circRIP assay was conducted as previously described [20]. Biotin-labeled circCRKL was synthesized by the Gene-Chem Company (shanghai, China), after which the products were extracted and tested using qRT-PCR.

### Luciferase reporter assays

The wild-type (wt) and mutated (mut) sequences of the circCRKL and CDKN1B 3ʹ untranslated regions (UTR) containing miRNA binding regions were amplified and cloned into a pmirGlo vector (Gene Pharma, Shanghai, China). A dual-luciferase assay was conducted as described [17].

### Western blotting analysis

The total proteins were extracted with RIPA lysis buffer, while bicinchoninic acid (Beyotime, China, Cat no. P0012-1) was used to detect the protein concentration. Western blotting analysis was performed as previously described [16], using p27 (Abcam, USA, Cat no. ab32034), GAPDH (Proteintech, USA, Cat no. 60,004-1-Ig), and HRP-conjugated secondary goat anti-mouse (Proteintech, USA, Cat no. SA00001–1) and goat anti-rabbit (Proteintech, USA, Cat no. SA00001–2) antibodies.

### Statistical analysis

An unpaired Student’s t-test was used to evaluate the group differences between normally distributed variables, while one-way analysis of variance was used for comparisons between three or more groups. The data were expressed as the mean ± standard deviation using GraphPad Prism 8.01 (GraphPad Prism Inc., La Jolla, USA) and SPSS 19.0 statistical software. *P* < 0.05 was accepted as statistically significant.

## Results

This study hypothesized that circCRKL inhibited AML cells proliferation and demonstrated that circCRKL was down-regulated in the AML samples and cell lines using qRT-PCR. A CCK8 assay, a colony-forming assay and cell cycle analysis were used to reveal the role of circCRKL in proliferation of AML cells. The relationship between circCRKL, p27, and miR-196a-5p/miR-196b-5p was predicted using bioinformatics tools and verified via luciferase assays. Furthermore, this study revealed that circCRKL inhibited AML cells proliferation by sponging miR-196a-5p/miR-196b-5p to affect p27 expression.

### CircCRKL is down-expressed in AML samples and cell lines

**Figure 1.**
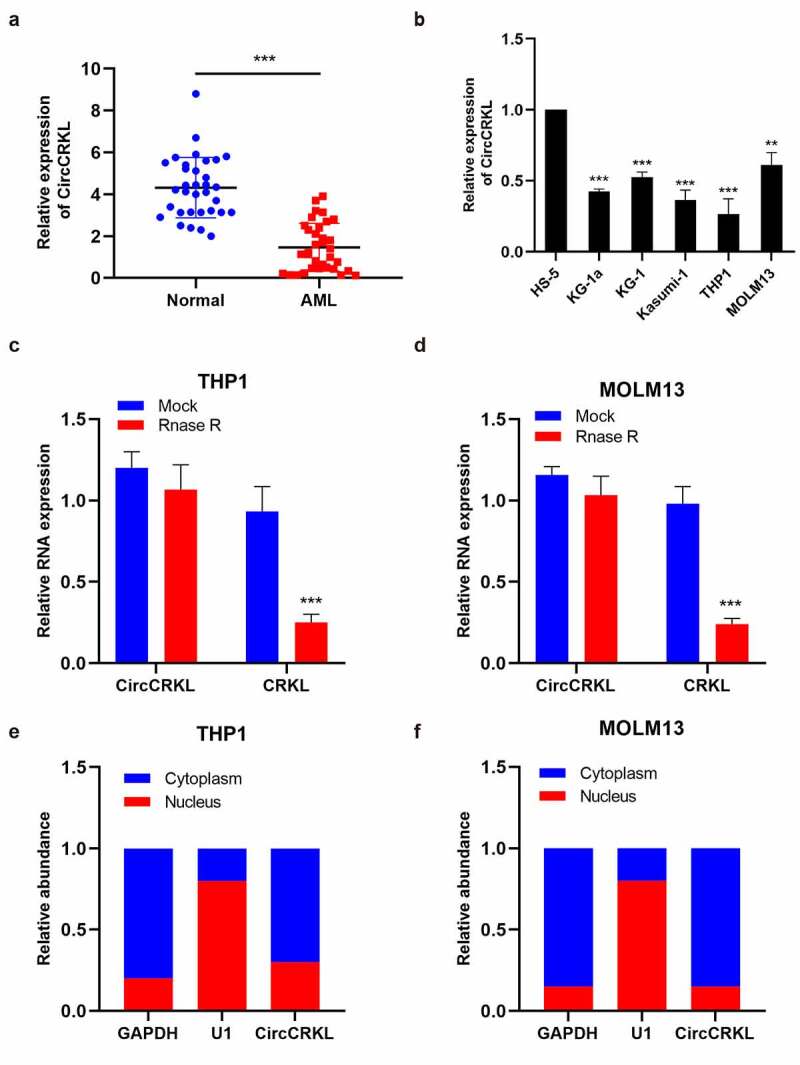
CircCRKL is down-expressed in AML samples and cell lines. (a) the expression of circCRKL in the AML samples (N = 34) and normal healthy samples (N = 34). (b) the expression of circCRKL in the HS-5 and AML cell lines (KG-1a, KG-1, kasumi-1, THP1 and MOLM13). (c, d) qRT-PCR analysis of the circCRKL expression in THP1 and MOLM13 cells after RNase R treatmet. (e, f) the circCRKL distribution was identified via the RNA nucleus/cytoplasm separation assay. **P < 0.01, ***P < 0.001

The expression of circCRKL in 34 clinical AML samples and normal samples (n = 34) was determined using qRT-PCR to assess the role of circCRKL in AML. (Figure 1(a)) shows that circCRKL was down-expressed in AML samples compared with the control samples. Then, the expression of circCRKL in human AML cells (KG-1a, KG-1, Kasumi-1, THP1, and MOLM13) and HS‐5 cells was analyzed using qRT-PCR. The AML cells exhibited lower circCRKL expression than the HS‐5 cells (Figure 1(b)). Since MOLM13 and THP1 expressed the highest and lowest level of circCRKL respectively, in the five AML cell lines, we conducted over-expression researches with THP1 and down-regulation with MOLM13. An RNase R assay was performed to determine the stability of circCRKL. Furthermore, CRKL mRNA expression displayed a significantly higher decline in the RNase R treatment group than in the Mock control group, while circCRKL was resistant to RNase R (Figure 1(c,d)). Furthermore, an RNA nucleus/cytoplasm separation assay was performed to identify circCRKL localization in the AML cells. The results indicated circCRKL enrichment in the cytoplasm (Figure 1(e,f)). Therefore, the expression and characteristics of circCRKL in the AML cells were preliminarily identified.

### CircCRKL suppresses cell proliferation in AML cells

**Figure 2.**
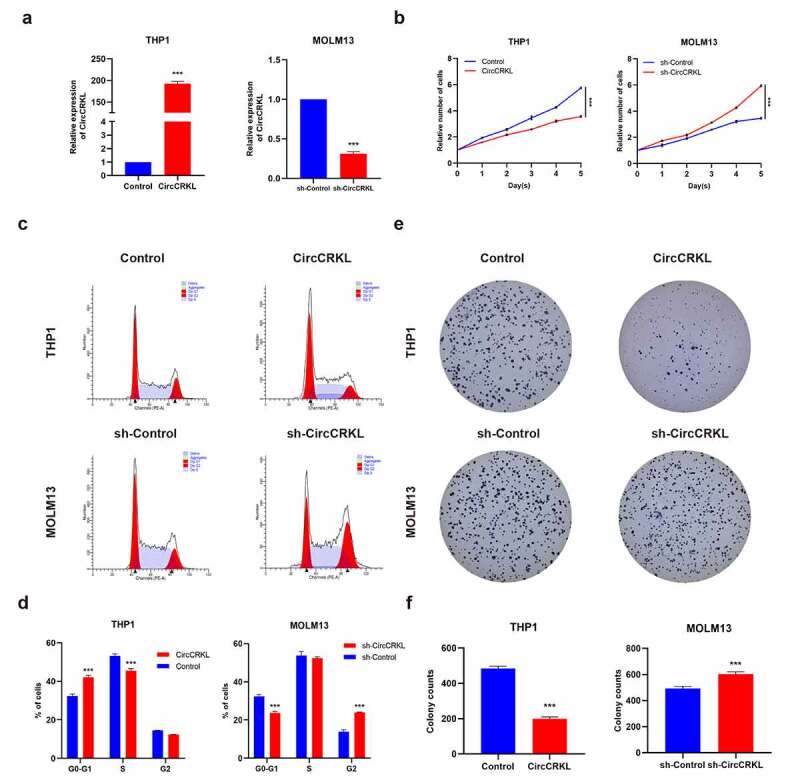
CircCRKL suppresses AML cell proliferation. (a) the expression of circCRKL using qRT-PCR. (b) A CCK-8 assay was used to detect the proliferation in transfected THP1 and MOLM13 cells. (c, d) the cell cycle distribution in transfected THP1 and MOLM13 cells were presented via flow cytometry. (e, f) the colony formation assay of the THP1 and MOLM13 cells transfected with circCRKL and sh-circCRKL, respectively. ***P < 0.001

CircCRKL was overexpressed in THP1 cells and downregulated in MOLM13 cells to explore its role in AML cells (Figure 2(a)). The CCK8 assay suggested that the growth rate decreased in the overexpressed circCRKL group, while silencing circCRKL expression promoted cell proliferation (Figure 2(b)). The cell cycle assay showed that increased circCRKL expression induced cell cycle arrest, while circCRKL down-regulation promoted cell cycle progression (Figure 2(c,d)). Although circCRKL reduced the colony-forming capacity of AML cells, silencing circCRKL expression increased this ability (Figure 2(e,f)). These results indicated that circCRKL inhibited AML cell proliferation and cell cycle progression.

### CircCRKL suppresses AML cells proliferation through directly interacting with miR-196a-5p and miR-196b-5p

**Figure 3.**
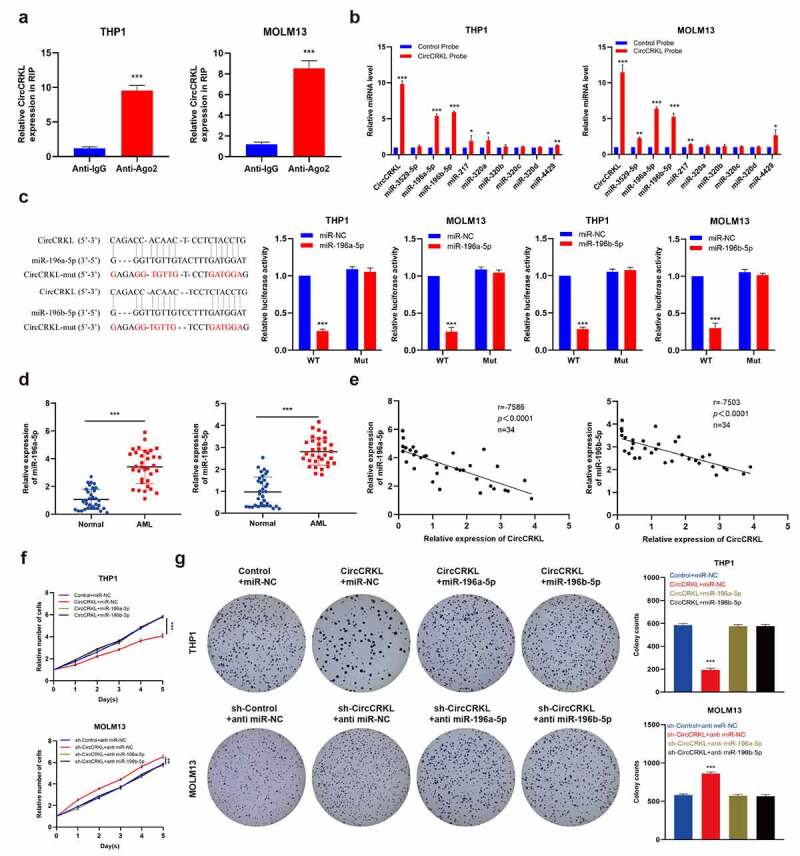
CircCRKL suppresses AML cell proliferation by directly interacting with miR-196a-5p and miR-196b-5p. (a) the RIP assay of circCRKL. (b) the circRIP assay was performed using circFGFR1 and NC probes. (c) A schematic showing the predicted circCRKL and miR-196a-5p/miR-196b-5p binding sites, as well as the mutation of the potential miRNAs-binding sequences in circCRKL. the luciferase activity in the AML cells was co-transfected with a luciferase reporter containing either circCRKL-wt or circCRKL-mut and miR-196a-5p/miR-196b-5p mimics. (d) MiR-196a-5p and miR-196b-5p expression in the AML samples. (e) the correlation between the miR-196a-5p/miR-196b-5p expression and circCRKL levels. (f) the CCK-8 assay of the indicated AML cells. (g) the colony formation assay of the indicated cells. **P < 0.01, ***P < 0.001

Studies have shown that sponge miRNAs facilitate circRNA functionality and that circCRKL is mainly distributed in the cytoplasm. Therefore, an RIP assay was performed to elucidate whether circCRKL acted as a miRNA sponge to enable its functionality. The results showed that the circCRKL was significantly enriched by the AGO2 antibody (Figure 3(a)), indicating that its incorporation into the RNA-induced silencing complex. Next, the starBase [21] was used to predict the potential binding between the miRNAs and circCRKL. The top nine miRNA (clipreadNum scores>1000) candidates were screened according to their clipreadNum scores, while the circCRKL-related RNAs were purified using RIP assays. The nine miRNA candidates were then analyzed via pull-down assays. Compared with the control, circCRKL and miR-196a-5p/miR-196b-5p were specifically enriched, indicating that miR-196a-5p and miR-196b-5p were the critical circCRKL-associated miRNAs in AML. (Figure 3(b)). A luciferase assay verified that miR-196a-5p and miR-196b-5p were targeted by circCRKL (Figure 3(c)). Next, the relationship between circCRKL and miR-196a-5p/miR-196b-5p in AML was explored further. Compared with the normal group, the miR-196a-5p and miR-196b-5p levels were up-regulated in the AML group (Figure 3(d)), which was negatively correlated with circCRKL (Figure 3(e)). The CCK8 assay and colony forming assay results further indicated that enforced expression of miR-196a-5p and miR-196b-5p effectively abolished the tumor-inhibiting effect of circCRKL in AML cells (Figure 3(f,g)). These findings showed that miR-196a-5p and miR-196b-5p were targeted by circCRKL and could reverse its impact in AML cells.

### P27 is regulated by miR-196a-5p and miR-196b-5p and inhibits cell proliferation

**Figure 4.**
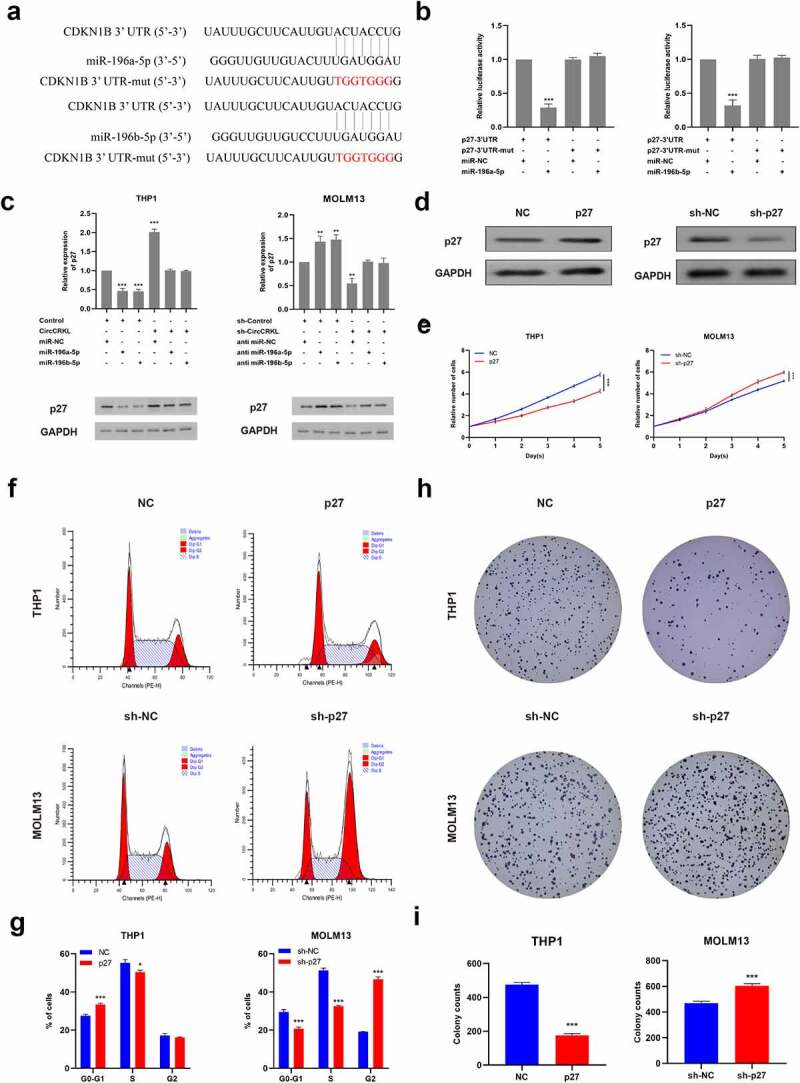
P27 is regulated by miR-196a-5p and miR-196b-5p and inhibits cell proliferation. (a) the p27-3’ UTR sequences containing miR-196a-5p and miR-196b-5p binding sites. (b) the luciferase activity in the AML cells co-transfected with a luciferase reporter containing either p27 3’ UTR-wt or p27 3’ UTR-mut and miR-196a-5p/miR-196b-5p mimics. (c) the p27 expression levels in the THP1 and MOLM13 cells transfected with miR-196a-5p/miR-196b-5p mimics alone or co-transfected with circCRKL or sh-circCRKL. (d) the p27 protein expression in the AML cells. (e) the CCK-8 assay of AML cells. (f, g) the cell cycle assay of the AML cells. (h, i) the colony formation assay of the AML cells. *P < 0.05, **P < 0.01, ***P < 0.001

Two bioinformatics algorithms, namely TargetScan [22] and miRDB [23], were used to determine that miR-196a-5p and miR-196b-5p targeted p27, which acted as a cyclin-dependent kinase inhibitor to mediate cell-cycle restriction. This was also consistent with our previous experimental results, revealing that circCRKL induced cell cycle arrest. Furthermore, luciferase assay results confirmed that p27 was, in fact, directly targeted by miR-196a-5p and miR-196b-5p (Figure 4(a,b)). Further evaluation indicated that miR-196a-5p and miR-196b-5p significantly reduced p27 expression, which could be reversed by circCRKL (Figure 4(c)). Then, p27 was up-regulated or down-regulated in THP-1 and MOLM13, respectively, via transfection to further explore its functionality (Figure 4(d)). The CCK-8, colony-forming and cell cycle assay results demonstrated that p27 inhibited cell proliferation by inducing cell cycle arrest, while p27 expression silencing promoted cell proliferation via cell cycle progression(Figure 4(e-i)).

## Discussion

AML is considered a deadly hematopoietic malignancy. Despite advances in AML therapeutic regimens, approximately half of patients experience recurrence or resistance and die of the disease. Therefore, the identification of new therapeutic targets is urgently needed. With the emergence and development of high-throughput sequencing technologies, more and more circRNAs have been found to be abnormally expressed in carcinomas and other diseases [24]. In addition, they also participate in many cellular biological processes, such as the migration, invasion, differentiation, proliferation and angiogenesis of cancer cells. CircRNAs have been proven to participate in AML progression. Sun et al. revealed that circMYBL2 promoted the progression of AML by regulating post-transcriptional translation [10]. Moreover, circSPI1 acts as an oncogene in AML [25]. This study investigated the role of circCRKL in AML. The results demonstrated that circCRKL inhibits AML cell proliferation, providing further evidence regarding the role of circCRKL in AML.

Some circRNAs contain miRNA response elements that can be used as ceRNA to sponge miRNAs, regulating downstream gene expression and affecting the biological functionality of diseases. For example, circ_0001162 is involved glioma development via the miR-936/ERBB4 axis [19]. CircRNA_0082835 promotes primary melanoma progression by sponging miRNA-429 [26]. However, these studies mainly focused on solid tumors. Here we first revealed that circCRKL inhibited the proliferation of AML cells by sponging miR-196a-5p and miR-196b-5p. First, circCRKL was distributed predominantly in the cytoplasm, indicating that it occupied the same space as miRNAs. Second, the expression of circCRKL was negatively correlated with miRNA expression. In addition, the results showed that circCRKL and p27 3′UTR displayed identical miR-196a-5p/miR-196b-5p bite sites. In summary, this study revealed a circCRKL/miR-196a-5p/miR-196b-5p/p27 axis function in AML.

P27, encoded by the CDKN1B gene, is a member of the kinase inhibitory protein (Kip) family and a cyclin-dependent kinase inhibitor that mediates cell-cycle inhibition. The CDKN1B gene encoding human p27 is involved in regulating the cyclin E/cyclin-dependent kinase 2 (CDK2) complex [27]. Since cancer cells can proliferate indefinitely, the levels and activity of cell cycle-related proteins, including p27, have attracted considerable attention. Accordingly, cancers with abnormal p27 metabolism/localization display a poor prognosis and response to treatment. Studies have shown that post-translational modifications, such as amino acid phosphorylation, primarily affect the functionality and cellular localization of the p27 protein [28]. However, p27 protein expression can be regulated via p27 modulation at the transcriptional and post-transcriptional levels. This study indicated that circCRKL interacted with miR-196a-5p and miR-196b-5p to increase p27 expression, providing more evidence for the post-transcriptional modification of p27 via circRNAs. Research has also shown that p27 is closely related to AML prognosis [29]. This work confirmed that p27 inhibited AML cells proliferation. Furthermore, circCRKL restricted the growth of AML cells by promoting p27 expression.

However, this research still presents some limitations. According to reports, p27 is also involved in tumorigenesis via the independent cyclin-cdk pathway [30]. Studies have also reported that p27 inhibited promoter transcription of target genes, such as AURKA and MED18, which are closely related to the poor tumor prognosis [31,32]. Therefore, it is not clear whether p27 performs functions in other pathways, and the downstream targets of p27 require further study. In addition, additional *in vivo* experiments are necessary to support the conclusions of this study.

## Conclusion

This work demonstrates that circCRKL is down-regulated in AML, showing potential as a therapeutic target for AML, while it can sponge miR-196a-5p and miR-196b-5p to inhibit p27 expression. This research explored the potential mechanism of circCRKL functionality in AML cells and provided a potential target for AML treatment.

## Supplementary Material

Supplemental Material
