# Supplementary material for: Circular RNA circCRKL inhibits the proliferation of acute myeloid leukemia cells via the miR-196a-5p/miR-196b-5p/p27 axis
Source: Bioengineered. 2021 Oct 7;12(1):7704–13. doi: 10.1080/21655979.2021.1982310 (PMC8806729; doi:10.1080/21655979.2021.1982310)
Supplement: Supplemental Material [file KBIE_A_1982310_SM6615.zip › supplementary/Additional file 1.docx]

Table S1: Characteristics of AML patients used in this study.

| Characteristics | AML (n=34) |
| --- | --- |
| Age at diagnosis (y, Median (range)) | 50.5 (16-74) |
| Sex |  |
| Male, n | 16 |
| Female, n | 18 |
| FAB |  |
| M0, n | 1 |
| M1, n | 7 |
| M2, n | 3 |
| M4, n | 3 |
| M5, n | 18 |
| M6, n | 2 |
